# Serving on a Graduate Medical Education Diversity, Equity, Inclusion, and Justice Committee: Lessons Learned From a Journey of Growth and Healing

**DOI:** 10.3389/fpubh.2022.867035

**Published:** 2022-04-27

**Authors:** Areeba Kara, Curtis Wright, Levi Funches, Francesca Williamson, Ralph A. Hicks, Timothy A. Sutton, Zeina Nabhan

**Affiliations:** Indiana University School of Medicine, Indianapolis, IN, United States

**Keywords:** graduate medical education (GME), diversity and inclusion, rapid qualitative methods, minority tax, committee

## Abstract

Efforts toward achieving diversity, equity, inclusion, and justice (DEIJ) within graduate medical education (GME) often begin with the formation of a DEIJ committee that steers the work. Little is known about the experiences and the challenges faced by those serving on such committees. We sought to describe the experiences of members of our institutional GME DEIJ committee to gain knowledge that would propel this work forward. An open-ended survey was electronically administered to members of our institutional GME DEIJ committee. Responses were analyzed using a rapid qualitative analytical approach. Eighteen members (58%) responded. Of these, (67%) were women and five (28%) were Black. Six domains emerged: “motivation,” “challenges,” “emotional response,” “highs,” “facilitators,” and “advice.” Black respondents more often cited the need to increase diversity as a motivator to join this work. Women and Black respondents more often identified time constraints as a challenge to participation. Some members found the work emotionally draining; others described it as uplifting. Two themes emerged as high points of participation-pride and achievement around the work completed and the personal benefits of building a community with a shared purpose. Three themes emerged as facilitators: effective leadership, support, and establishing psychological safety during the meetings. Many arrived at the realization that change would take time and advocated for patience and perseverance. Protected time and DEIJ expertise were identified as integral to successful committee work. Our findings provide novel insights into the experience of serving on a GME DEIJ committee and highlights infrastructural and institutional prerequisites for success.

## Introduction

Confronted with evidence of race-related disparities in health care, institutions across the country are working to recognize, describe, and dismantle discriminatory infrastructures (1, 2). Diversifying the healthcare workforce is a strategy to decrease healthcare disparities, and it is the crucial responsibility of graduate medical education (GME) programs to train the leaders, researchers, educators, advocates, and caregivers in medicine for each generation (3). GME however may have its own unique iterations of structural racism which contribute to the underrepresentation of Black and Latinx members in the physician workforce. Overall, in 2020 in the United States, only 7.8% of physician residents were Hispanic while only 5.8% were Black (4). These deficits are amplified across the trajectory of an academic career with even lower proportions of Hispanic and Black faculty members represented within the ranks of associate and full professors (5).

There is an increasing recognition for the need to transform rapidly and many GME sites have created programming to improve the way they deliver on their responsibilities. These efforts are further spurred by new common program requirements mandated by the Accreditation Council for Graduate Medical Education (ACGME) requiring GME programs to report on their recruitment and retention practices to enhance diversity (6). Many such initiatives begin with the formation of a committee which then steers the work forward. While guidance exists in the literature to foster effective committee work, less is known about how committee members themselves perceive this work. Committees formed to advance diversity, equity, inclusion, and justice (DEIJ) efforts may face distinct challenges related to the complexity, sensitivity, and emotions inherent in the subject.

Our goal was to describe the experiences of the members of a GME DEIJ committee. These descriptions can help us learn how to foster psychologically safe environments, anticipate threats to success, and maximize the creativity and productivity that is needed to move this work forward.

## Methods

### Setting

Our institution, located in the Midwestern US, is one of the largest medical schools and GME sites in the country. We encompass 102 ACGME-accredited programs, collectively graduating more than 1,200 trainees annually. The GME DEIJ committee was launched in August 2020 with a call for volunteers. The final membership comprised of 22 faculty members representing 17 different specialties, six residents/fellows, a program coordinator, administrator, and project manager. Monthly virtual meetings commenced in October 2020. The committee was charged by the Designated Institutional Officer with developing programs and processes to address the DEIJ needs of our GME programs and simultaneously fulfill the ACGME core program requirements surrounding diversity and health care disparity teaching.

### Survey

To gain insights into committee members' experiences, we created and administered an open-ended survey (Appendix). Questions addressed the motivation to join the committee, challenges and fulfillment originating from membership and the facilitators and barriers to work done. The authors, many who are experienced in qualitative research, discussed the survey to assure face validity. The survey was created in REDCap- a secure, web-based application designed to support data capture for research studies (7). The survey was sent electronically to all members in September 2021. There were no incentives for survey completion and results were anonymous. The survey remained open for 2 weeks with two reminders sent during that time.

### Analysis

All authors participated in analysis. Survey response transcripts were downloaded from REDCap. We used a rapid qualitative deductive analysis approach to understand the experience of participants (8). We first created a neutral domain name to correspond with each question in the survey. Based on these domains a template was created to summarize responses. The summary template included a section for “other” observations for material that did not match the identified domains and a section to capture important quotations. Each member of the author team then used the template to summarize the same transcript. The team then met to assess consistency and the usability of the template. Once consistency was established and the template was refined, transcripts were divided among the team for summarization. These synopses were then used to generate a matrix (respondent type compared to the domain). “Matrices streamline the process of noting simultaneously and systematically similarities, differences, and trends in responses across groups of informants” (9). The matrix was used to consolidate responses within domains by finding themes, and to compare similarities and differences between respondent types. The team met collectively to iteratively identify, consolidate, and refine domains and themes and to develop an understanding of the experiences described and we are confident that the results reflect these experiences.

## Results

A total of 18 committee members responded to the survey (response rate 58%) and offered important insights. Two-thirds of respondents were women and half were <40 years old. More than a quarter identified as Black or as immigrants (*n* = 5, 27.8% for both) (Table 1).

**Table 1:** Demographics of respondents.

**Characteristics**	***N* (%)**
**Total**	18
**Self-identified gender**
Woman	12 (66.7%)
Man	6 (33.3%)
**Age (years)**	
20–30	1 (5.5%)
31–40	8 (44.4%)
41–59	6 (33.3%)
51–60	2 (11.1%)
≥61	1 (5.5%)
**Race and ethnicity** ^*^	
Black	5 (27.8%)
White	9 (50%)
Asian	3 (16.7%)
Latinx	2 (11.1%)
Not stated	2 (11.1%)
**Immigrant**	5 (27.8%)

* *Respondents were asked to check all that apply*.

Participants shared their experiences within six domains including “motivation,” “challenges,” “emotional response and management,” “highs,” “facilitators,” and “general advice.” Within each domain, certain themes emerged and are described further below and in Table 2.

**Table 2:** Domains, themes and representative quotes emerging from analysis.

**Domain**	**Themes**	**Representative Quotations**
**Motivation**		
	Systemic	“Want[ed] the staff and leadership environment for GME to look more like the population.”
	Personal	“the opportunity to learn about other's experiences with diversity issues and their dedication to combat it.”
**Challenges**		
	Systemic	“[There is] the feeling that we should be part of a bigger campus-wide movement, but the silos that helped get us where we are still very much exist, preventing progress.”
	Personal	“...Introspection was difficult”, “time”
	Subject matter related	“..doing anything to improve DEI will be an uphill struggle”
	Process related	“The remote meeting format was a limitation….”
**Emotional response and management**		
	Draining	“this role is very emotionally taxing. We got to hear about most of the racist things that occur in our community and relive some of our own trauma.”
	Uplifting	“Overall, I think this role was emotionally therapeutic. It feels good to help create change.”
	Coping	“I am able to manage because I feel like we are making great progress and have a dedicated committee.”
**Highs**		
	Pride in achievements	“Highs were … seeing our ideas come to fruition.”
	Building a community	“Having a group who feels as strongly that there is a need for changes and hearing others' ideas”
**Facilitators**		
	Strong leadership	“Our leadership during this period was outstanding. Meetings with open dialogue were wonderful and creating concrete next steps made the process meaningful and engaging.”
	Institutional support	“The support of GME leadership was key.”
	Psychological safety	“the committee team members who are passionate about this work”
**Advice**		
	Systemic	“Incentivizing gains in DEI will involve both financial and time commitments from the institution.”
	Personal	“be patient -DEIJ work takes time to actualize.”
	Process related	“I believe you have to choose one or two things and concentrate on them”

### Motivation

Respondents described multiple motivators that prompted their decision to join this committee. These motivators often related to the dual desires of seeking improvements within the **system** and/or improvements and fulfillment in oneself. In describing the need for improvements within the institution, participants often cited the recognition of the lack of diversity on campus as a key motivator. One respondent noted that they “Want[ed] the staff and leadership environment for GME to look more like the population.” Creating an institutional environment where those underrepresented in medicine would thrive was motivating for others- with participants driven by either their own personal negative experiences, or those they had witnessed among trainees. Involvement in DEIJ efforts as part of a personal journey of growth and learning was the driving force for some. One respondent described how “current events [COVID-19, the murder of George Floyd and other Black individuals], my coincidental reading and self-education about racism in our history” led to joining the committee. The desire for self-improvement through positive contribution and learning from others was described by some members. As one participant noted they were “Tired of not being part of the solution for my peers” while another embraced “the opportunity to learn about other's experiences with diversity issues and their dedication to combat it.”

In examining the responses within matrices, the impetus to participate in the committee being tied to the desire to increase diversity and decrease healthcare disparities was more frequently described by Black committee members while personal evolution or interest was more frequently described by White participants.

### Challenges

Respondents described the obstacles to participating fully in the work of the GME DEIJ committee as revolving around four themes- these challenges were often related to **systemic** issues, **personal** issues, issues **inherent to the topic** they were tackling or related to the **process/logistics**—specifically the inability to gather in person due to pandemic related constraints.

Challenges at the system level were identified by some. One participant desired integrated efforts across the enterprise. They articulated—“[There is] the feeling that we should be part of a bigger campus-wide movement, but the silos that helped get us where we are still very much exist, preventing progress.”

Personal challenges to participation were frequently described as the dearth of time and the absence of dedicated or protected time for engaging in the work. Indeed, time was repeatedly cited as a limitation with many describing discouragement around the disparity between how much they wanted to contribute compared to how much they could, due to lack of time. A unique personal challenge was the self-examination that resulted from participation in the committee. One member noted “I too had hurdles as the first to graduate college in my family, but there are so many more hurdles out there that many face. Becoming educated in that regard was eye opening and humbling. I thought I came from a pretty unbiased perspective, but that was not true. Introspection was difficult.” Some respondents questioned the worth of their contribution with one being hampered by “the internal feeling of not having anything of substance to add to the conversation.”

Still others found the magnitude of the task at hand overwhelming and were burdened by the realization of the multiple barriers to progress. As one stated “Knowing that because we live in a very socially/politically conservative state, doing anything to improve DEI will be an uphill struggle.” Another noted how—“listening to some of the discussions about diversity, the unfairness and mistreatment of others” was difficult.

The virtual format was felt by some to inhibit effective work—as one described “The remote meeting format was a limitation, but it was great that it was still an avenue.”

In exploring differences in responses by respondent type, it was noted that women, and those who identified as Black invoked the lack of time as a constraint more frequently than those who were men or White.

### Emotional Response and Management

While some participants described participation as emotionally draining, others described it as uplifting. Those describing emotional taxation often reported despondency with the current state. As one participant noted “There was a sense of urgency and responsibility that at times weighed heavy.” Those who found it uplifting often invoked the satisfaction derived by being involved in positive change. As one noted “Overall, I think this role was emotionally therapeutic. It feels good to help create change.” Black respondents bore the additional burden of personally identifying with the issues discussed. One noted “this role is very emotionally taxing. We got to hear about most of the racist things that occur in our community and relive some of our own trauma.”

Most respondents were unable to identify strategies they used to mitigate negative emotions and cope. Some described that focusing on progress and the long-term commitment of the work was helpful. As one stated, “I am able to manage because I feel like we are making great progress and have a dedicated committee.”

### Highs

Two themes emerged as high points of participation- there was a sense of **pride and achievement** around the work completed and the personal benefits of **building a community** with a shared purpose. Committee members described pride in the committee's tangible achievements including creating a mission and vision statement and a toolbox for best practices for recruitment. One noted “Highs were … seeing our ideas come to fruition.” The sense of community uplifted others and they reported joy in finding a group of like-minded individuals to learn alongside and with whom they shared a vision. As one described this “Having a group who feels as strongly that there is a need for changes and hearing others' ideas” was a high point.

### Facilitators

In describing what facilitated effective committee work three themes emerged: the importance of effective **leadership, support**, and the **psychological safety** perceived during meetings. The engagement, open communication style of the committee leaders was appreciated as was the commitment articulated for this work by leaders in GME. The ability to share ideas openly and freely without fear of judgment was cited as an important facilitator. One respondent described “Our leadership during this period was outstanding. Meetings with open dialogue were wonderful and creating concrete next steps made the process meaningful and engaging.”

### Advice

We solicited advice from survey respondents on how to be effective in DEIJ efforts. The resulting suggestions fell into three categories: **systemic**, **personal** or **process**-oriented.

Some offered advice for systems including the need to engage and support these efforts continuously—“Incentivizing gains in DEI will involve both financial and time commitments from the institution.”

On a personal level, many arrived at the realization that change would take time and advocated for patience and perseverance. One described “actionable change will take a long time” and another exhorted “be patient -DEIJ work takes time to actualize.” The need to engage in DEIJ efforts and grow continuously as a personal responsibility was emphasized- “Just be kind to everyone! Treat others the way you want to be treated.” Personal growth, education and responsibility was also specifically described in finding the courage to become an upstander—“it is never too late to intervene when you see something wrong. Tell the person at the moment, but if it is a week later, because you had to process or get the courage, then do it then too and take time to educate yourself on the history of racism in medicine.”

Specific suggestions on how to operationalize these processes were offered by some including the need to prioritize and focus efforts and to seek advice from experts. One noted “I believe you have to choose one or two things and concentrate on them” and another responded “Do not payroll initiatives without clear deliverables. Hire experts in the field to augment the work of the invested faculty, residents, and students.”

## Discussion

These exploratory findings provide us with novel insights into the experience of serving on a DEIJ committee within a GME program and have implications for the infrastructural and institutional support that may be needed to facilitate the success of DEIJ efforts.

A committee is formed to address a specific series of tasks or goals. Its members and leaders can facilitate great accomplishments or promote stagnation. Much has been written about what constitutes a successful team across a multitude of disciplines, and several models of team effectiveness have been proposed (10–16). While a review of all aspects of team building is beyond the scope of this work, a few principles are worth highlighting. An effective committee should have a clear purpose, informal climate, robust participation, open communication, shared leadership, and the ability to build consensus. The committee members should possess listening skills, style diversity, disagree with civility and collaborate/communicate effectively. The committee leader should define the team's purpose, clarify roles, establish meeting norms, encourage participation, share the limelight, celebrate accomplishments, facilitate team reflection on committee effectiveness, and foster a team-oriented culture for the organization (17).

Our findings reinforce the importance of each of these guiding principles and uncover other nuances that may be unique to DEIJ work. Committee leaders should anticipate the emotional toll that participants may experience and practice and model strategies for acknowledging, understanding, and managing this emotion. The motivators to join DEIJ efforts may be stronger than those that drive other initiatives- and may simultaneously and paradoxically facilitate rapid progress and rapid frustration. Focusing efforts and tackling a mixture of issues may be one strategy to harness the passion that members bring to such teams. Selecting initiatives that yield some quick victories while continuing to tackle long term goals may be beneficial. Setting expectations, anticipating timelines and periodically reminding the team of accomplishments may be especially important in DEIJ committees to maintain momentum and morale. Such guidance requires strong, empathetic, skillful leadership and great care should be exercised in appointing leaders of such work. Our recommendations are summarized in Table 3.

**Table 3:** Recommendations on the conduct of Diversity Equity Inclusion and Justice (DEIJ) committees.

**Before commencing**
Identify empathetic and skillful leadership with effective communication skills
Assure institutional commitment to the committee's overall mission and goals
Advocate for administrative and financial support including protected time
Secure the support and time of a DEIJ content expert
**As the work starts**
Anticipate emotions and model how to manage them
Develop strategies and opportunities to engage all committee members
Recognize and support the different motivators for participation
Choose a mixture of short, intermediate, and long-term goals
**As the work continues**
Periodically remind team members of progress
Work to build a community among the team
Acknowledge and mitigate the differential impact of volunteering time on women
and minoritized participants

Many participants voiced an insecurity about their expertise to handle DEIJ content. DEIJ work is specialized, sensitive and many dedicated scholars have devoted their careers to this work. For DEIJ committees to succeed, embedding such expertise within these teams is crucial. Embedded DEIJ experts can guide teams away from ideas that may be detrimental, hasten the adoption of proven methodology and assuage the anxieties of those who feel inauthentic in their expertise. Institutions should also provide adequate development support, financial resources, and structural power to DEIJ leaders and these groups so they have the autonomy and agency needed to effect change (18).

We gleaned additional important lessons that are relevant at the institutional level. DEIJ efforts cannot be successful in isolation and require an integrated approach between the missions of education, clinical practice, scholarship, research, and leadership. In the era of partnerships between academic institutions, health care practices, and hospitals, this work requires a cohesive and coordinated approach. Participants in our sample identified the silos between each entity as a threat to DEIJ. Support at the institutional level was also considered crucial to success- with this support being recognized in various forms. Leadership and administrative assistance and the provision of expertise were all appreciated- however time constraints were repeatedly cited as a bottleneck. Institutions must consider that protected time and effort may be necessary for faculty to engage fully. Time constraints were cited more often by women and minoritized faculty members. These comments are characteristic of the “minority tax,” a term used to describe how institutions—intentionally or unintendedly—often ask marginalized faculty to take on more responsibilities than their non-marginalized counterparts to achieve diversity goals (19). Institutions should work to reward DEIJ activities in meaningful ways and ensure broad participation in these efforts.

Our work has certain limitations and strengths. While our sample size is small, the views gathered were from diverse perspectives and were rich in detail. Our focus was on understanding the experiences of those participating in such committees and informing others who are engaged in these efforts; but there are many other aspects of DEIJ work that need further exploration. Future work may include eliciting the experiences of those not involved in such work and monitoring the evolution of perspectives over time. The differences we noted between respondent types should generate further conversations and study. The anonymity of the survey precluded the identification of non-responders, and we cannot present the views of those who did not respond.

While the ultimate goal of this work is to establish a culture of true DEIJ, we found some unexpected joys in the process. The COVID-19 pandemic has increased rates of burnout among healthcare workers- and many participants found solace in the creation of a community of like-minded individuals (20). The scholarship emerging from this work has also become a source of pride among the team with many participating enthusiastically despite the raging pandemic. As we work toward the difficult goals of achieving equity and justice for our faculty, communities, and patients, we must recognize and celebrate the unanticipated rewards along the way. We hope that the lessons learnt from this work will help advance committees focused on DEIJ efforts everywhere.

## Data Availability Statement

The raw data supporting the conclusions of this article will be made available by the authors, without undue reservation.

## Ethics Statement

Ethical review and approval was not required for the study on human participants in accordance with the local legislation and institutional requirements. Written informed consent for participation was not required for this study in accordance with the national legislation and the institutional requirements.

## Author Contributions

AK contributed to conception and design of the study and organized the database, and wrote the first draft of the manuscript. CW, FW, and ZN wrote sections of the manuscript. All authors performed the analysis and contributed to manuscript revision, read, and approved the submitted version.

## Conflict of Interest

The authors declare that the research was conducted in the absence of any commercial or financial relationships that could be construed as a potential conflict of interest.

## Publisher's Note

All claims expressed in this article are solely those of the authors and do not necessarily represent those of their affiliated organizations, or those of the publisher, the editors and the reviewers. Any product that may be evaluated in this article, or claim that may be made by its manufacturer, is not guaranteed or endorsed by the publisher.
