# Supplementary material for: Serving on a Graduate Medical Education Diversity, Equity, Inclusion, and Justice Committee: Lessons Learned From a Journey of Growth and Healing
Source: Front Public Health. 2022 Apr 27;10:867035. doi: 10.3389/fpubh.2022.867035 (PMC9091374; doi:10.3389/fpubh.2022.867035)
Supplement: Supplementary file 1 [file Data_Sheet_1.PDF]

Describing the experience of serving on a diversity committee

Thank you for your service on the GME Diversity Committee. To help us describe this experience, please complete the survey below.

1) Reflecting back to the time when you agreed to serve on the GME task force committee- please describe what motivated you to take on this effort. __________________________________

2) What was the hardest part of being a member of this committee for you? __________________________________

3) Please reflect on whether this role was emotionally taxing for you. If so, why, and how did you manage? __________________________________

4) What do you recall as the 'highs' and 'lows' of your participation in this committee? __________________________________

5) In your opinion, what were the facilitators and barriers that supported (or hampered) the work done in this committee? __________________________________

6) What advice would you give to others (or to IU) to be effective in their efforts to promote diversity, equity and inclusion? __________________________________

7) If you would be willing to talk to us further (or prefer to answer these questions in person) please provide your name and we shall reach out! Thank you!! __________________________________

8) Please select your age (in years)
- ◯ 20-30
- ◯ 31-40
- ◯ 41-50
- ◯ 51-60
- ◯ 61 or older
- ◯ Prefer not to answer

9) Please select the gender you identify with
- ◯ Woman
- ◯ Man
- ◯ Non-binary
- ◯ Transgender
- ◯ Prefer not to answer

10) Please select your race, ethnicity and immigrant status (select all that apply)
- ☐ White
- ☐ Black
- ☐ Latinx
- ☐ Immigrant
- ☐ Asian
- ☐ Prefer not to answer
